# Surgical Site Infections in Gastrointestinal Surgeries: Estimation of Prevalence, Risk Factors and Bacteriological Profile

**DOI:** 10.7759/cureus.62589

**Published:** 2024-06-18

**Authors:** Bargavi K, Secunda R, Saravanan J, Jeswanth Satyanesan

**Affiliations:** 1 Medicine, Stanley Medical College, Chennai, IND; 2 Surgical Gastroenterology, Stanley Medical College, Chennai, IND

**Keywords:** healthcare associated infection, antimicrobial resistance, infection control, gastrointestinal surgery, surgical site infection (ssi), patient safety, nosocomial infection, esbl, antibiotic resistance

## Abstract

Introduction: Surgical site infections (SSIs) are one of the leading causes of operation-related adverse effects. To create an effective hospital infection program, information about a local pattern is essential. The ever-changing pattern of infections and inappropriate use of antibiotics has predisposed to the development of drug-resistant strains and has made the management of SSIs arduous.

Aims and objectives: The purpose of this study is to estimate prevalence and identify risk factors and commonest organisms associated with SSIs.

Methodology: In this analytical cross-sectional study, the relationship between various risk factors and the development of SSIs was evaluated in patients undergoing elective surgery and staying at least seven days postoperatively in the Department of Surgical Gastroenterology and Liver transplant for a study duration of two months. Diagnosis of SSIs was based on modified CDC criteria. Wound pus was followed by blood collection in suspected secondary septicemia. MacConkey and blood agar were used to culture pus; brain heart infusion broth was used for blood samples. Antimicrobial susceptibility testing was carried out using Mueller-Hinton agar by the Kirby-Bauer method.

Results: Twelve out of 50 had developed postsurgical wound infections where Gram-negative organisms prevailed over Gram-positive ones. The associated risk factors identified in this study are age, BMI, wound class, American Society of Anesthesiologists (ASA) score, preprocedural WBC count >10,000, and the duration of surgery. Escherichia coli is the causative microbe for the majority of infections (35.7%). Gram-negative bacteria isolated in this study were extended-spectrum β-lactamase (ESBL) producers. Multidrug-resistant organisms were predominant.

Conclusion: The present study identified an SSI rate of 24% in gastrointestinal surgeries. The sensitivity and resistance pattern of the organisms isolated will help in measures to be taken to devise a proper and effective current hospital antibiotic prophylaxis policy.

## Introduction

Postoperative wound infections also known as surgical site infections (SSIs) are the most common source of preventable mortality and morbidity. According to the National Nosocomial Infections Surveillance (NNIS) system, SSIs are the third most frequently reported nosocomial infections, accounting for 14%-16% of all nosocomial infections among hospitalized patients [1,2]. SSIs tend to complicate the recovery of patients forcing them to incur financial burdens for prolonged hospital stays and might have some unfavorable consequences like untimely demise. 

According to the CDC, SSIs can be defined as an infection that is present up to 30 days after a surgical procedure that is devoid of implants at the site or part of the body where the surgery took place and up to one year if an implantable device was placed in the patient. However, 50% develop in the first week and almost 90% of SSIs manifest within the second week of surgery. The causative pathogens depend on the type of surgery as well as the demography; the most commonly isolated organisms are Escherichia coli, Enterococcus faecalis, and Pseudomonas aeruginosa in gastrointestinal surgeries [3]. SSIs may be classified as superficial/incisional limited to the skin and subcutaneous tissue, deep incisional when involving the fascia and muscle, or organ space when involving a body cavity (e.g. abdominal cavity following gastrointestinal surgery). Deep tissue and organ space SSIs are less frequently encountered than superficial SSIs but are associated with greater morbidity/mortality, readmission rates, longer hospital stay, and increased overall hospital-associated costs when compared with superficial SSIs. Although the majority of SSIs are uncomplicated, others may be severe and more challenging to manage, such as necrotizing deep soft tissue infections. The latter often require extensive surgical debridement and multiple reoperations and may even be life-threatening. Minimizing SSIs is the supreme concern for surgeons and hospitals for patients undergoing surgery (both elective and emergency). 

SSIs are the index of the health care system of any hospital. For surgical patients, SSIs are the most common nosocomial infections and they are the leading cause of operation-related adverse events. Infected wounds might prolong hospital stay for about 5 to 20 days and can cause a significant increase in medical expenses incurred by the patient [4]. The mortality rate with SSIs is 3% and the cause of mortality can be traced back to SSIs in 75% [1]. In this context, it becomes important to determine the prevalence of surgical site infections, take into consideration the magnitude of the problem, and provide a reason to start taking serious measures on infection control in hospitals. Many developing countries and ones with fewer resources have inefficient hospital infection control programs. While developed countries have been successful in decreasing the rates of SSI due to advances in infection control practices such as ensuring proper ventilation in operating rooms, sterilization techniques, and hand hygiene practices, countries with limited health budgets show higher rates [5]. The ever-changing pattern of hospital-acquired infections (HAIs) and inappropriate use of antibiotics has predisposed to the development of drug-resistant strains and has made the management of SSIs arduous. With the increase in the incidence of nosocomial infections and multi-drug resistance, meticulous and periodic surveillance of various HAIs is called for. The identification of SSIs involves careful evaluation of clinical and laboratory findings, and programs to monitor infections must use standardized terminology; otherwise, discrepant SSI rates will be computed and reported [6]. To create an effective hospital infection program, information about local patterns is essential. This type of information may be valuable to the control of infections in hospitals and prove to be insightful at a national level.

## Materials and methods

This analytical cross-sectional study includes 50 participants. Here, the prevalence of SSIs and microbial isolates and the relationship between various risk factors contributing to SSIs were studied over a period of two months in (June-July) 2019 at the Department of Surgical Gastroenterology and Liver Transplant after obtaining approval from the Institutional Ethics Committee. Informed consent was obtained from those who came under the inclusion criteria from this study. Patients who underwent elective surgeries and stayed for at least seven days postoperatively were included in the study. Grossly contaminated wounds and diagnosis of definitive cellulitis which may be treated with antibiotics by itself do not meet the criteria to be included as superficial SSIs under part 'd' of the definition mentioned in NHSN Surgical site infection event of the CDC [1], immunosuppressed participants or those consuming immunosuppressant medications like steroids and improperly collected and labeled specimens were excluded from this study. Patients who returned to the theatre due to complications following earlier surgery can be included only if the index procedure is not already included. Patients were excluded if the primary indication for surgery was gynecological, obstetric, urological, or transplantation because the gastrointestinal tract is not typically opened.

A detailed history of the patient from preoperative to postoperative measures was collected. Data collection included patient-level factors such as age, sex, socioeconomic details, nutritional status, smoking history, and alcohol consumption; comorbidities such as diabetes, hypertension, chronic obstructive pulmonary disease, renal insufficiency, history of use of immunosuppressant medication; operative level factors such as American Society of Anesthesiologists (ASA) score, wound class, type of surgery, duration of surgery, history of previous blood transfusion and blood loss. The primary outcome measure was the 30-day SSI incidence (defined by US CDC criteria). When an SSI was suspected, a swab was collected from the site and if the presence of an SSI was confirmed, the type of SSI, date of onset, and microorganism(s) cultured were reported. 

The criteria for diagnosis of SSIs are discussed below [1].

Superficial SSI

The date of the event occurs within 30 days and involves only skin and subcutaneous tissue of the incision and has one of the following. Purulent drainage /organism(s) are identified from an aseptically obtained specimen from the superficial incision or subcutaneous tissue by a culture/localized pain or tenderness, localized swelling, erythema, or heat.

Deep SSI

The date of the event occurs within 30 or 90 days and involves deep soft tissues of the incision and one of the following: purulent drainage from the deep incision/a deep incision that is deliberately opened or aspirated and organism(s) identified from the deep soft tissues of the incision by culture or nonculture based microbiologic testing method and fever (>38°C), localized pain or tenderness/an abscess or other evidence of infection detected on anatomical, histopathologic, or imaging test.

Organ/space SSI 

The date of the event occurs within 30 or 90 days and involves any part of the body deeper than the fascial/muscle layers that are opened or manipulated during the operative procedure and one of the following: purulent drainage from the drain placed into organ or space/ organisms are identified from fluid or tissue in the organ or space/ evidence of infection on anatomical, histopathological, or imaging test.

Wound pus was collected before dressing using a sterile swab. Excess pus is collected in a sterile syringe. The swab and the pus in the syringe were cultured on Sterile MacConkey agar and blood agar followed by overnight incubation at 37°C. Blood samples obtained by venipuncture were inoculated in brain heart infusion broth and incubated at 37°C for five days. Every 24 hours, the bottle was inspected for turbidity, and a subculture was made on sterile MacConkey agar and blood agar by the quadrant streaking method. The obtained colonies on MacConkey agar and blood agar were subjected to Gram’s Staining, biochemical tests of identification, and antibiotic susceptibility test (Kirby-Bauer method) on Mueller-Hinton agar. 

Data processing and statistical analysis of the risk factors for patients were done using IBM SPSS Statistics for Windows, Version 26 (Released 2019; IBM Corp., Armonk, New York, United States) [7]. Frequency and percentage analysis were used for categorical variables; mean and standard deviation were used for continuous variables. With a 95% confidence interval, a p-value of less than 0.05 was considered statistically significant.

## Results

Age and sex distribution

Out of the 50 participants, the mean age of participants was found to be 42.71 years with a standard deviation of 19.16. The maximum was 76 years while the minimum was 11 years. Table 1 shows the age distribution of the participants. From participants with less than or equal to 20 years, three developed SSIs (42.85%); from 21 to 35 years, two participants developed SSIs (22.23%); from 36 to 50 years, four participants developed SSIs (26.67%); from 51 to 65 years, two participants developed SSIs (18.18%); and from those above 65 years, one developed an SSI (12.5%). About 68% (n=34) of the participants of the study were male out of which 11 developed SSIs (32.35), and the remaining 32% (n=16) were female out of which one developed an SSI (6.25%). 

**Table 1:** Age categories and sex distribution of the participants who developed SSIs (N=50) SSIs: Surgical site infections

Age distribution	Number of participants (n)	Number of participants infected (n)
≤ 20	7	3
21-35	9	2
36-50	15	4
51-65	11	2
>65	8	1
Sex distribution of participants	Male	34
	Female	16

Type of infection 

The overall rate of infection was found to be 24%(n=12) with superficial incisional infections being 75% and deep incisional infections being 25% (Table 2). Superficial incisional infections involve only the skin and subcutaneous tissue. They were suspected when one of the findings was present: purulent discharge, at least one symptom of infection or discharge yielding a positive culture. These are the most common type of SSI. Deep incisional infections involve deeper tissues like muscles and facial planes. They were suspected when one of the following was present: purulent discharge, wound dehiscence, evidence of abscess formation, or deliberate reopening of the incision by the surgeon after suspecting an infection. The mean days after which SSIs were detected in the current study was 6.25.

**Table 2:** Type of infection among study participants who developed SSIs (N=50) SSIs: Surgical site infections

Participants	Frequency (n)	Type of infection	Frequency (n)
Infected	12	Superficial SSI	9
		Deep SSI	3
Not infected	38		

Wound class 

The Center for Disease Control and Prevention replaced the SWC system created to represent the bacterial load and classified wounds into four different classes [8]. Class 1 includes clean wounds that are not infected and do not exhibit any signs of inflammation. They don't involve a breach in the respiratory, alimentary, or urogenital tract. Class 2 are classified as clean/contaminated with a low level of contamination as they involve entry into the respiratory, alimentary, genital, or urinary tracts but only under controlled conditions. Class 3 are contaminated wounds typically resulting from a breach in sterile techniques or leakage from the gastrointestinal tract. Class 4 are considered to be dirty or infected with an active infection in the surgical site with a purulent exudate, surgery with fecal contamination or a ruptured viscus. Out of participants with wound class 1, 2 out of 5 developed SSIs (40%); with wound class 2, 3 out of 35 developed SSIs (8.57%); with wound class 3, 6 out of 9 developed SSIs (66.67%); and with wound class 4, one patient developed an SSI (Table 3). 

**Table 3:** Wound class of participants and the frequency of participants with SSIs (N=50) SSIs: Surgical site infections

Wound class	Description	Total (n)	Participants with SSIs (n)
1	Clean	5	2
2	Clean/contaminated	35	3
3	Contaminated	9	6
4	Dirty/Infected	1	1

Identified pathogens

Out of the 12 patients who had positive cultures, 10 patients were infected with a single pathogen (83.34%) and two patients had mixed cultures (16.66%): MRSA and E. coli, Klebsiella and Pseudomonas. Escherichia coli was the commonest species isolated (5 out of 12 cultures) from culture accounting for 35.7%, followed by Klebsiella with 4 out of 12 cultures, accounting for 28.5% (Table 4). 11 out of 12 cultures were from Gram negative microbes (91.67%) and 1 out of 12 cultures was mixed with the Gram-positive isolate being MRSA and the Gram-negative isolate being E. coli. Two out of the 12 participants had developed secondary septicemia as represented in Table 5. 

**Table 4:** Pathogens associated with SSIs among study participants (N=50) SSIs: Surgical site infections

Organism	Number (n)	Percentage (%)
Escherichia coli	5	35.7
Klebsiella	4	28.5
Pseudomonas	2	14.2
Acinetobacter	2	14.2
MRSA	1	7.1

**Table 5:** Pathogens isolated from secondary septicemia participants (N=50)

Secondary septicemia participants	Correlation between samples	
	Wound	Blood
Participant 1	Klebsiella	Klebsiella
Participant 2	E. coli and MRSA	Staphylococcus aureus

Antimicrobial susceptibility pattern of the isolates

The tested antibiotics include aminoglycosides (Amikacin and Gentamycin), cephalosporins (Cefipime, Cefatoxime and Ceftazidime), quinolones (Ciprofloxacin, Norfloxacin, Levofloxacin), carbapenems (Meropenem, Imipenem), and tigecycline for Gram-negative bacteria. Penicillin, ampicillin, bacitracin, vancomycin (minimum inhibitory concentration for Staphylococcus aureus), doxycycline, teicoplanin and linezolid for a Gram-positive bacterium. The Gram-negative organisms isolated in the study showed high resistance to Ciprofloxacin (100%) and Amoxicillin-clavulanate (100%) as seen in Table 6. E. coli, the commonest isolate (5 out of 12), was highly resistant to all tested antibiotics except tigecycline (100% sensitive), amikacin (60% sensitive) and gentamycin (20% sensitive). Klebsiella was 100% sensitive (2 out of 2 isolates) to tigecycline and imepenem and 50% sensitive (1 out of 2) to gentamicin, amikacin, meropenem, and cotrimoxazole. The Gram-positive isolate, MRSA was sensitive to vancomycin, doxycycline, teicoplanin and linezolid as shown in Table 7.

**Table 6:** Antimicrobial susceptibility patterns of gram-negative bacterial isolates from wound cultures of participants suspected to have SSIs (N=12) IMP: Imepenem, TGC: tigecycline, GEN: gentamycin, AK: amikacin, FEP: cefipime, CAZ: ceftazidime, MRP: meropenem, CIP: ciprofloxacin, LEV: levofloxacin, TAX: cefotaxime, TMP-SMX: cotrimoxazole, PTZ: piperacillin tazobactam, AMC: amoxiclav, SSIs: surgical site infections.

Organism isolated	Sample	IMP	TGC	GEN	AK	FEP	CAZ	MRP	CIP	LEV	TAX	TMP-SMX	PTZ	AMC
Pseudomonas	pus	R	R	S	S	S	R	S	R	R	R	R	S	R
Acinetobacter	pus	R	S	R	R	R	S	R	R	R	R	R	R	R
Klebsiella	Pus – mixed growth of Klebsiella and Pseudomonas	R	S	R	R	R	R	R	R	R	R	S	R	R
Pseudomonas		R	R	S	S	S	R	S	R	S	S	R	S	R
Klebsiella	serous discharge	R	S	R	R	R	R	R	R	R	R	R	R	R
Escherichia coli	pus	R	S	R	R	R	R	R	R	R	R	R	R	R
Acinetobacter	serous discharge	R	S	S	R	R	R	R	R	R	R	R	R	R
Escherichia coli	serous discharge	R	S	R	S	R	R	R	R	R	R	R	R	R
Escherichia coli	pus	R	S	R	R	R	R	R	R	R	R	R	R	R
Escherichia coli	pus	R	S	R	S	R	R	R	R	R	R	R	R	R
Klebsiella	pus	S	S	S	S	R	R	S	R	R	R	S	R	R
Escherichia coli	pus	R	S	S	S	R	R	R	R	R	R	R	R	R
Klebsiella	pus	S	S	R	R	R	R	R	R	R	R	R	R	R

**Table 7:** Antimicrobial susceptibility patterns of gram-positive bacterial isolates from wound cultures of participants suspected to have SSIs SSIs: Surgical site infections

Antibiotic tested	Susceptibility pattern of MRSA isolated to antibiotics
Penicillin	R
Ampicillin	R
Bacitracin	R
Vancomycin	S
Doxycycline	S
Teicoplanin	S
Linezolid	S
Cotrimoxazole	R
Cefoxitin	R
Erythromycin	R

Relationship of SSIs with risk factors

A detailed history of the patient from preoperative to postoperative measures were collected and then followed up periodically. The following information was looked upon: Patient level factors such as age, sex, socioeconomic details, BMI, alcohol consumption and smoking; co-morbidities such as previous history of diabetes mellitus, hypertension, chronic obstructive pulmonary diseases, renal insufficiency and history of recent use of immunosuppressants; operative level factors such as ASA score, wound class, type of surgery, duration, blood loss during the surgery and history of transfusions; and other environmental factors (Table 8). Among the factors analyzed, the duration of surgery, ASA score, wound class, and preprocedural WBC count emerged as statistically significant risk factors for the development of SSIs.

**Table 8:** Association between the risk factors that emerged to be significant and SSIs among the study participants (N=50) SSIs: Surgical site infections; ASA: American Society of Anesthesiologists

Risk factor	Parameter	Infected (n)	Not infected (n)	p-value
Surgery duration	<2 hours	9	16	0.047
	>2 hours	3	22	
ASA score	1	0	1	0.042
	2	0	8	
	3	11	28	
	4	1	1	
Wound class	1	2	3	0.01
	2	3	32	
	3	6	3	
	4	1	0	
Preprocedural WBC count	< 10,000	5	29	0.026
	>10,000	7	9	

Pattern and duration of procedures

**Figure 1:**
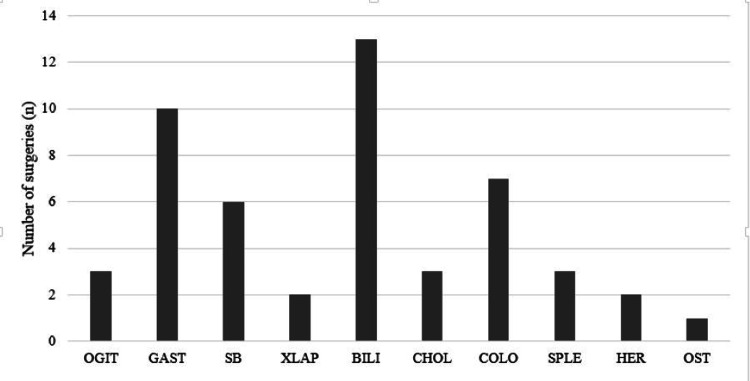
Pattern of gastrointestinal procedures done during the period under study (N=50) OGIT: Other gastrointestinal procedures, GAST: gastric, SB: small bowel, XLAP: laparotomy, BILI: liver/pancreas/biliary tract, CHOL: cholecystectomy, COLO: colorectal, SPLE: splenectomy, HER: hernia repair, OST: ostomy reversal

**Figure 2:**
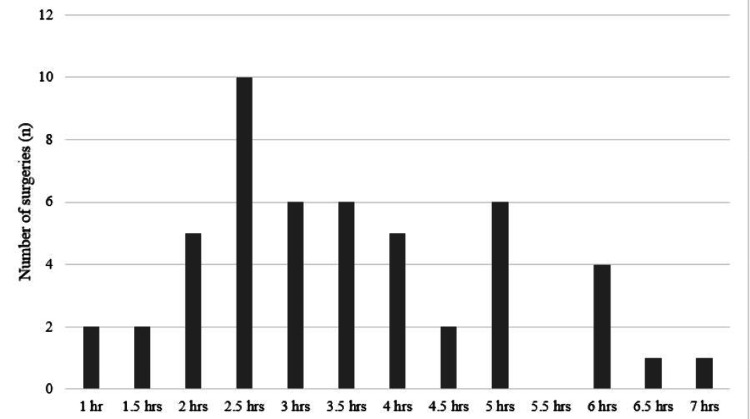
Duration of surgery for the procedures done during the period under study (N=50)

The duration of surgery was defined as the time from skin incision to that of closure of wound. The shortest duration recorded was one hour and the longest duration was seven hours. Among the classified procedures, surgery of the liver, pancreas and biliary tract accounted for the majority (26%) followed by gastric surgery (20%) (Figure 1). Nine out of 50 (18%) surgeries lasted for a duration of less than or equal to two hours; 31 out of 50 surgeries lasted for a duration for greater than two hours (62%) (Figure 2). The mean duration of surgery was 3.51 hours. All infected surgeries had an operative duration of equal to or greater than two hours.

## Discussion

The rate of SSIs as predicted by the current study is 24%. Even though it is significantly higher than the rates quoted in surgical literature, it is in concordance with the rates seen throughout India. The higher rates in this study could be due to the pre-existing infections and co-morbidities in the patients included [9].

To identify the risk factors that lead to the development of SSIs, various parameters were analyzed statistically. The results of logistic regression analysis showed that SSIs were related to the age, nutritional status of the patient, ASA score, wound class, preprocedural WBC count as well as the duration of surgery. The age of the patient emerged as a risk factor (p=0.046). The highest rates were seen among the age range of 21-50 as shown in Table 1. The rate of infection is proportional to an increase in age especially after 50 years of age due to a waning immune system, existing co-morbidities, and poor response to stressors a major reason for which is delayed or impaired wound healing. In the current study, males were at a greater risk of developing SSIs. The nature of the relationship between sex and SSI is unclear and is yet to be brought to light as the number of male and female participants wasn't the same.

In the current study, both underweight (BMI<18.5) and overweight (BMI>25) have been associated with postsurgical wound infection (p=0.032). Malnourished patients have a compromised immune system which can be rectified by the provision of nutritional support to the patient before surgery; this is said to be effective in reducing the risk of infections, especially in colorectal cancer [9]. In obese individuals, mobility is compromised hence the reduced skeletal muscle mass. This is further compounded by inadequate tissue perfusion providing insufficient nutrition required for tissue repair [10].

Regarding the administration of prophylactic antibiotics before surgery, ideally, they should be administered 30-60 minutes before the incision and should be administered consistently [1]. For surgical prophylaxis, antibiotics with the lowest antimicrobial spectrum are advisable to prevent the development of multi-drug resistance. The level of antimicrobial should be maintained throughout surgery. A second dose is indicated in conditions of severe blood loss (>1500 ml blood) or when the duration of surgery is prolonged. Blood transfusions due to their immunomodulatory effect might account for the development of SSIs in some cases with associated comorbidities. Duration of the surgery, when lasts for more than two hours, has been proven to be an independent risk factor for the development of infection(p=0.047) [11]. The ASA score (p=0.042) and wound class (p=0.01) are directly proportional to the development of SSIs. ASA scores of II and III have been proven as independent risk factors for the development of SSIs [12]. Reduction in the risk of SSIs in patients with an ASA score of 4 or 5 is attributed to a minority of the population classified that way parallel to the discrepancies encountered at times due to variable wound class in this study.

The type of anesthesia depends on the age, procedure performed, complexity of the procedure, and preference. Some studies demonstrate an increased rate of SSIs with the use of general anesthesia; the current study shows no significant relationship between general versus regional anesthesia and the development of wound infection. Uncontrolled hyperglycemia is often associated with fluid and electrolyte disturbances and impaired host defenses like decreased polymorphonuclear cell mobilization. Since short-term control of blood glucose levels and perioperative blood glucose levels have been established as reliable factors for assessing the development of infections, parameters for long-term control of glucose like a history of diabetes mellitus or HbA1c might not be reliable indicators [13].

Secondary bacteremia is a condition wherein microbes enter the bloodstream as a complication of infections. Postoperative bacteremia developed in two cases and it has been shown in some studies that Staphylococcus aureus, isolate or growth in mixed culture is associated with twice the risk [14]. Bloodstream infections of such origin are associated with a poor prognosis. Escherichia coli is the commonest organism isolated (35.7%) as in Table 4 and, notably, the majority of the organisms isolated are Gram-negative this data is in agreement with other studies conducted [15], leading to surmise that the source of infection might have its roots from the patient’s endogenous microbiome colonizing the gut. Any event that topples the balance of oxidation and reduction in tissues may show promising growth of anaerobes. A potential cause of infection could be the spillage of gut contents during surgery or the endogenous flora from the patient’s skin [16]. Gram-negative bacteria isolated in this study were extended-spectrum β-lactamase (ESBL) producers. Klebsiella has shown increased resistance in the current study with 50 percent of the isolates exhibiting carbapenem resistance. The recent trend is the development of CRE, owing to their ability to collect resistance plasmids [17]. All the Gram-negative isolates were sensitive to tigecycline. The two Acinetobacter spp. isolated in this study showed multi-drug resistance with sensitivity only to tigecycline and colistin. Quinolones are starkly ineffective [18]. MRSA isolated has shown resistance to beta-lactam antibiotics and is sensitive to linezolid and teicoplanin as represented in Table 7.

Limitations of the study

As this study was a cross-sectional study, causality can't be proved between the risk factors assessed and the development of SSIs even though an association exists between said risk factors and SSIs. Other well-known factors having a known association with SSIs such as preoperative preparation by shaving and preoperative antibiotic use were not considered in the study. Emergency procedures were excluded from the study. The number of infected cases was small and this single-centered study showed a predominance of Gram-negative pathogens. A multi-centric approach with a larger study sample may be used to increase the power of the study and thus give an insight into the patterns and risk factors that are associated with the development of SSIs in the study population. Furthermore, incidence rate calculations may not be effectively extrapolated to an entire year for calculations as the data provided is for a period of two months that does not take into account environmental factors and other confounding variables. Since some variables had to be determined retrospectively with chart review, some variables could not be definitively ascertained.

## Conclusions

The present study identified an SSI rate of 24% in abdominal surgeries and also provides information on the risk factors associated with its occurrence. The rates were higher among the older age group, those with existing co-morbidities, wound class, and complicated surgeries with a prolonged duration of surgery. Hence, maintaining an aseptic environment in operation theatres should be emphasized and periodic assessment of the resistance patterns to the commonly used antibiotics is highly recommended. The choice of antibiotic prophylaxis should be dictated by the type of surgery and pathogens most likely to cause SSIs. In the current study, the most common organism isolated was Escherichia coli (35.7%). The sensitivity and resistance pattern of the organisms isolated in this study along with the sensitivity patterns of the predominant pathogen of this study will help in measures to be taken to devise a proper and effective current hospital antibiotic prophylaxis policy.
